# Obstacles and opportunities in Chinese pharmaceutical innovation

**DOI:** 10.1186/s12992-017-0244-6

**Published:** 2017-03-24

**Authors:** Jingyun Ni, Junrui Zhao, Carolina Oi Lam Ung, Yuanjia Hu, Hao Hu, Yitao Wang

**Affiliations:** Institute of Chinese Medical Sciences, State Key Laboratory of Quality Research in Chinese Medicine, University of Macau, Room 2053, N22, Avenida da Universidade, Taipa, Macau China

**Keywords:** China, Pharmaceutical industry, Obstacles, Opportunities, Global network, Innovation

## Abstract

**Background:**

Global healthcare innovation networks nowadays have expanded beyond developed countries with many developing countries joining the force and becoming important players. China, in particular, has seen a significant increase in the number of innovative firms and research organizations stepping up to the global network in recent years. Nevertheless, the intense Research and Development input has not brought about the expectable output. While China is ascending at a great speed to a leading position worldwide in terms of Research and Development investment, scientific publications and patents, the innovation capabilities in the pharmaceutical sector remain weak.

**Discussion:**

This study discusses the challenges and opportunities for pharmaceutical innovation in China. One hand, academic, industrial, institutional and financial constraints were found to be the major and inevitable barriers hindering the development of drug innovation. On the other hand, unique advantages had been observed which included growing pharmaceutical market, Research and Development funding, distinctive source, and international cooperation.

**Summary:**

The most important thing for China’s pharmaceutical sector to leap forward is to break though innovation barriers and integrate own advantages into global value-chain of healthcare product development.

## Background

Pharmaceuticals are playing an extreme important role in global health system by diagnosing, curing, treating, and preventing diseases. In terms of dramatically increasing R&D (Research and Development) expenditures and relatively decreasing approvals of new drugs during recent past, the decline in R&D efficiency has been the central issue of discussing global pharmaceutical innovation [1, 2]. Meanwhile, recent literature clearly points out that emerging countries mainly involving China show the increasing importance of pharmaceutical R&D activities and investments in innovative research for developing new drugs with the influence of R&D globalization [3, 4]. In this context, it is of great significance to understand pharmaceutical innovation in China from the global perspective.

As one of the fastest growing markets among the emerging countries, China received increasing attention from around the world. Due to supporting national polices, economic growth, aging population and global trend, China’s share of pharmaceutical industry output increased nearly seven‐fold, from 2.5% in 1995 to 18.3% in 2010, and is expected to become the second-largest pharmaceutical market in the world by 2020 [5, 6]. This changing trend may also apply to the global healthcare innovation networks as increased sales performance can better support R&D.

It is obvious that China has ascended to a worldwide leading position at an accelerated pace in terms of R&D funding, scientific publications, and patents in recent years [4]. With the perspective of switching from imitation to innovation, R&D expenditure in China’s pharmaceutical industry increased from $162.6 million USD (USA dollar) in 2000 to $3249.2 million USD in 2011 [7]. The favorable condition created by the tremendous investments made by Chinese pharmaceutical sector in R&D has resulted in significant global share of scientific publications and patents in recent years. The number of articles published by Chinese scholars in peer review journals related to pharmaceuticals has leapt to the second position in the world [8].

However, China is still weak in developing real innovative medicines. Considerable pharmaceutical R&D input, scientific publications and patents in China have not yet translated into the ultimate outcome of innovative pharmaceutical products recognized globally. For a long time, pharmaceutical industry in China is known for its mass-production of low-level generic drugs and as a ‘world factory’ of active pharmaceutical ingredients (APIs) with little mentioning of real innovative medicines [9]. Studies have shown that China remains at a weak position in the global drug innovation network based on analysis of worldwide recognized innovative drugs [10–12].

It is no doubt that the pharmaceutical innovation system in China is filled with obstacles which prevent China’s R&D capabilities from transforming into innovation competencies and eventually pharmaceutical products to generate market values [13]. With concerns about the huge gap between strong R&D input/ paper output as well as weak innovative medical products, this study aims to provoke a more systematic analysis of obstacles and opportunities in Chinese pharmaceutical innovation system. More understanding of pharmaceutical innovation system in China will be helpful to provide the more opportunity of discovering new medicines effectively in the world.

## Obstacles to pharmaceutical innovation in China

**Fig. 1:**
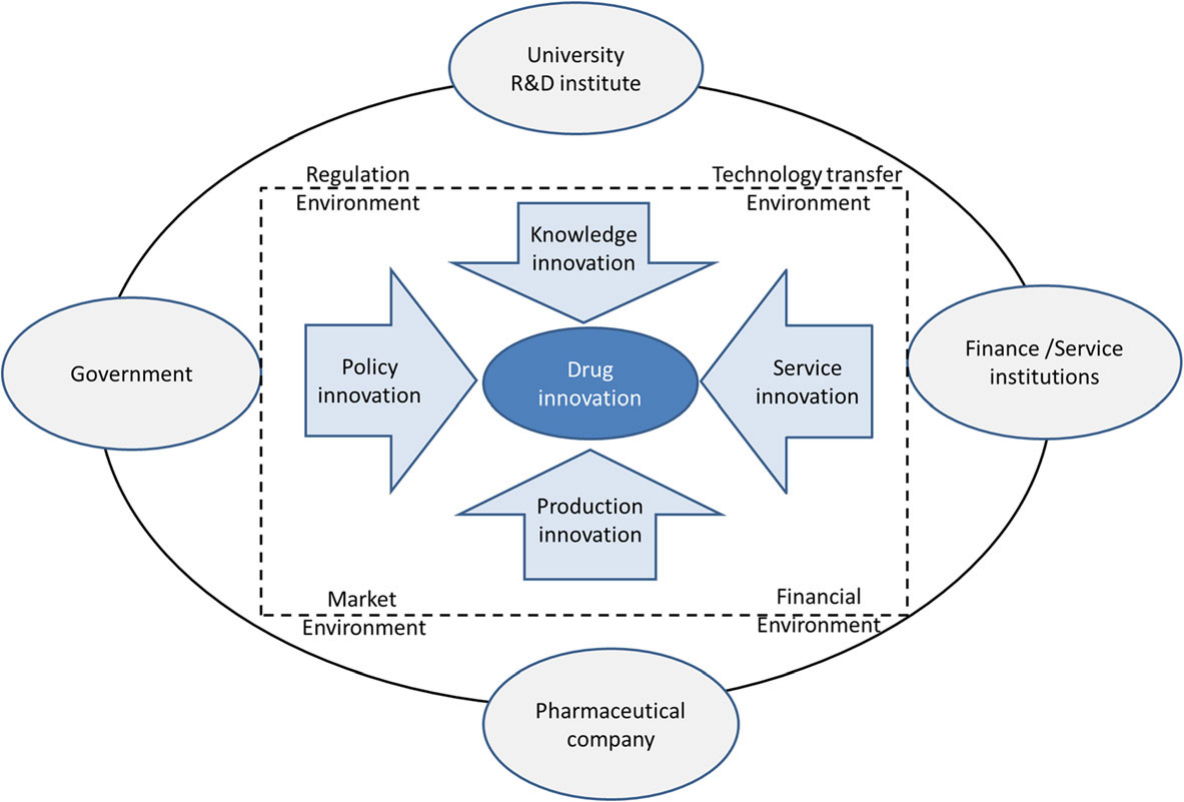
Pharmaceutical innovation system

Innovation is a system phenomenon, with multiple types of individual and collective agents, including firms, entrepreneurs, institutes for education and research, policymakers, regulatory agencies, and many types of services and intermediaries, interacting in a variety of ways [14]. Based on prior literatures [15, 16], Fig. 1 demonstrates pharmaceutical innovation system, which is comprised of R&D organizations, governments, pharmaceutical companies, finance and service institutions, responsible for knowledge innovation, policy innovation, production innovation, and service innovation, respectively. These innovations link together and generate new medicine discovery under a favorable regulation, market, finance, and technology transfer environments. Obstacles to pharmaceutical innovation in China have been observed at each of the above-mentioned counterparts which will be discussed further in the following.

### Academic organizations

It has been suggested that close partnerships among universities, institutions and companies are integral for the new business model of pharmaceutical R&D in China [17]. However, to maintain an effective collaboration between the science and the industry of pharmaceutical has always been challenging. Pharmaceutical researchers in universities and research institutes in China devote very much to the research work which does not usually take into consideration of the overall development of the pharmaceutical industry. As a result, the research work may not fully address and respond to the challenges and changing demands of the industry [18].

**Fig. 2:**
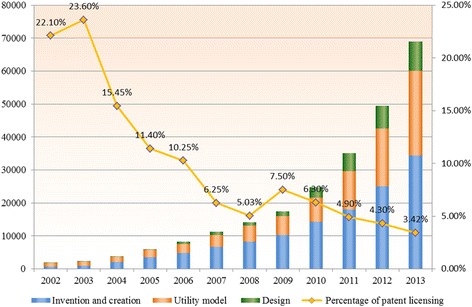
Granted patents and licensing percentage of Chinese universities. Data source: China Universities Statistics Yearbook

Moreover, paper output, i.e. scientific publications and patents, generated in the environment have been seriously criticized by international society, as is clearly shown in recent literatures [19–21]. The Science Citation Index (SCI) -based promotion scheme provides scholars with great incentives in terms of personal honors and has successfully encouraged them to produce a large quantity of publications and file many applications for patents. However, the citation rate of academic papers remains at a low level and the patent lives are short. As shown in Fig. 2, the proportion of licensed patents gradually declined, despite the rapid increase in the number of granted patents during the past decade. The difficulty of patent licensing by universities may imply a considerable gap between academic research and innovative products. Critics start to review the benefits and possible downside of the SCI-oriented research assessment criteria. It has been suggested that, under the scheme, scholars have become more oriented to personal achievements than the core value of research work, which has lowered innovation quality and slowed down the overall pharmaceutical innovation development in China [19, 20]. Commercialization of R&D output to real innovative drugs well thus falls behind.

### Pharmaceutical industry

In the context of industry, high fragmentation of the industrial structure, weak R&D intensity and serious product homogeneity are the major barriers to new drug development in China. As of 2012, there were around 4500 domestic pharmaceutical manufacturers and 14,000 domestic pharmaceutical distributors in China, which were attributed in three subsectors involving in chemical drug (50%), traditional Chinese medicines (32%) and biotechnology production (18%) [22].

**Fig. 3:**
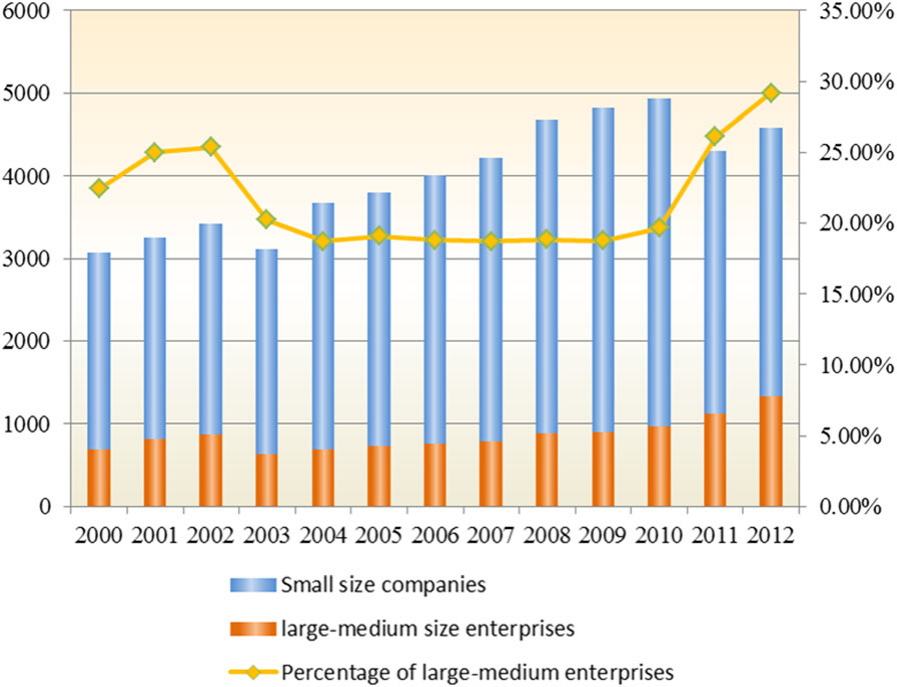
The number of pharmaceutical manufacture enterprises and percentage of large-medium enterprises in China. Data source: China High-tech Industry Statistics Yearbook

As shown in Fig. 3, more than 70% of pharmaceutical manufacturers are small-scale enterprises with employees less than 300 and operating revenue less than $3 million USD in China (according to China’s Regulations on Small and Medium- sized Enterprises (SMEs) Categorizing Criteria’ last accessed in 2011) [23]. It is difficult for them to sufficiently support R&D with all necessary financial resources to pursue new drug discovery.

**Fig. 4:**
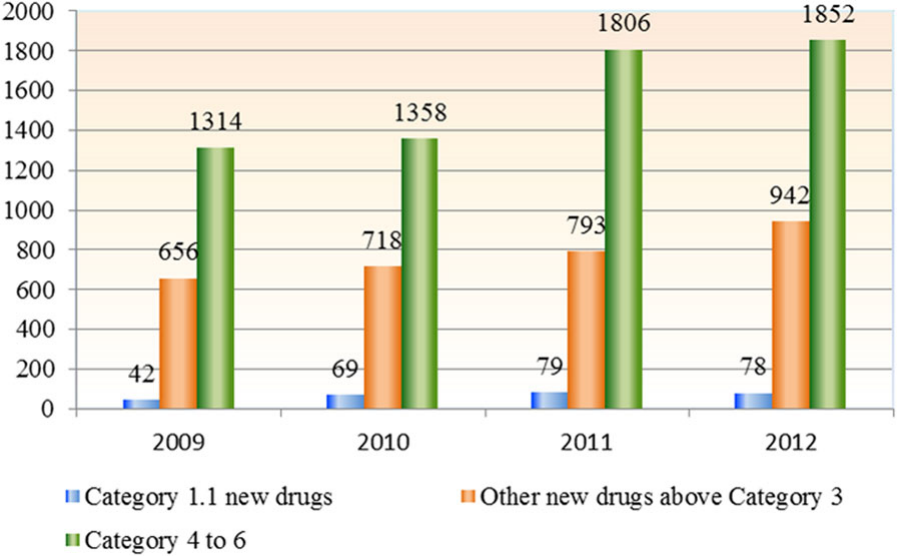
Number of chemical drug applications accepted by the CFDA from 2009 to 2012. 1. Data source: 2013 China Drug Review Annual Report. 2. Category 1.1 refers to new chemical drug which has never been previously approved for marketing as a drug anywhere else in the world. Category 3 of Chemical Drugs refers to a new drug which has only been marketed outside of China. Category 4 refers to Drug substance and its preparation with changed acid or alkaline radicals (or metallic elements), but without any pharmacological change, and the original drug entity already approved in China. Category 5 is defined as Drug preparation with changed dose form, but no change of administration route and the original preparation already approved in China. Category 6 refers to Drug substance or preparation following national standard

Meanwhile, current ratio of R&D investment to sales is about 2.7% in most of the Chinese pharmaceutical companies, which is significantly lower than that of US counterparts ranging from range of 15–20% [9, 24]. Due to lack of R&D resources for new drug discovery and development, most of the small-scale firms engaged mainly in low-value-added activities such as manufacturing, formulating, packaging and distributing generic products rather than innovation activities. At most, these pharmaceutical firms usually opted for developing generic drugs in order to obtain short-term revenue without going through the burden of high technical innovation. According to the ‘China Drug Review Annual Report’ released by the China Food and Drug Administration (CFDA) in 2012, the number of category 1.1 new drug applications which reflect the status of innovative drug development solely in domestic Chinese pharmaceutical companies remained around 70 per year over the past few years. On the contrary, applications of changing dosage form and new generic drugs accounted for more than 50% of chemical drug applications in China (see Fig. 4).

**Fig. 5:**
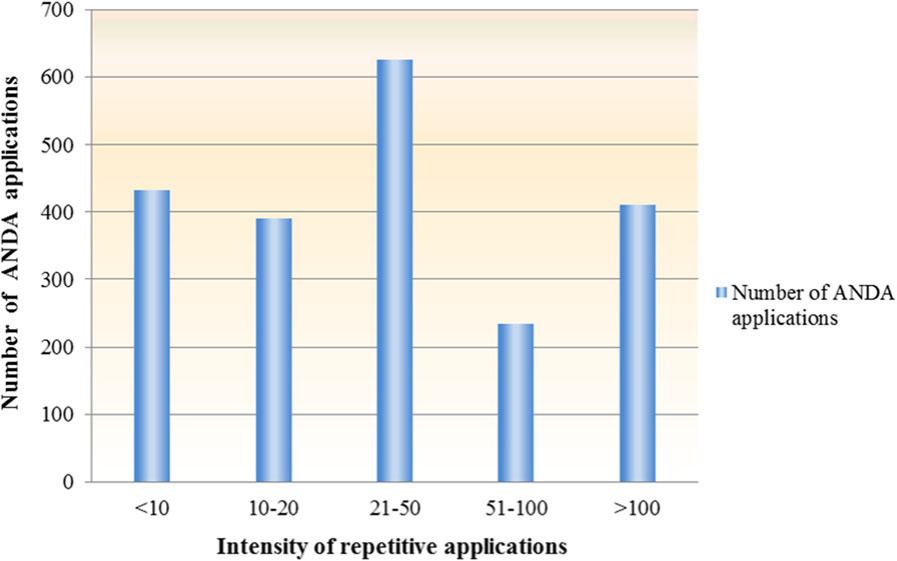
The distribution of ANDA applications. Data source: 2012 China Drug Review Annual Report

In addition, repetitive applications of generic drugs without high technical innovation became a prominent issue in the current pharmaceutical industry in China. Figure 5 indicates the distribution of the Abbreviated New Drug Application (ANDA) applications with existing approval numbers submitted in 2012. The vertical axis represents the number of ANDA applications, while the horizontal axis shows the intensity of repetitive applications. There were 1272 applications of generic drugs, each of which was repetitively submitted by different sponsors more than 20 times, accounting for 60.7% of the total in 2012. For example, in 2014, CFDA released the first list of overproduction drugs (more than 500), 34 categories of drugs are manufactured by more than 500 pharmaceutical companies in China, such as aspirin, ibuprofen, metronidazole, norfloxacin and so on.

The excessive development of homogeneous generic drugs resulted in over-capacity of the same products, which catalyzed the unordered market competition. While many manufacturers produced the same type of generic drugs, each manufacturer incurred only single-digit profit margin or might even experience financial loss [25].

### Regulation and administration

**Fig. 6:**
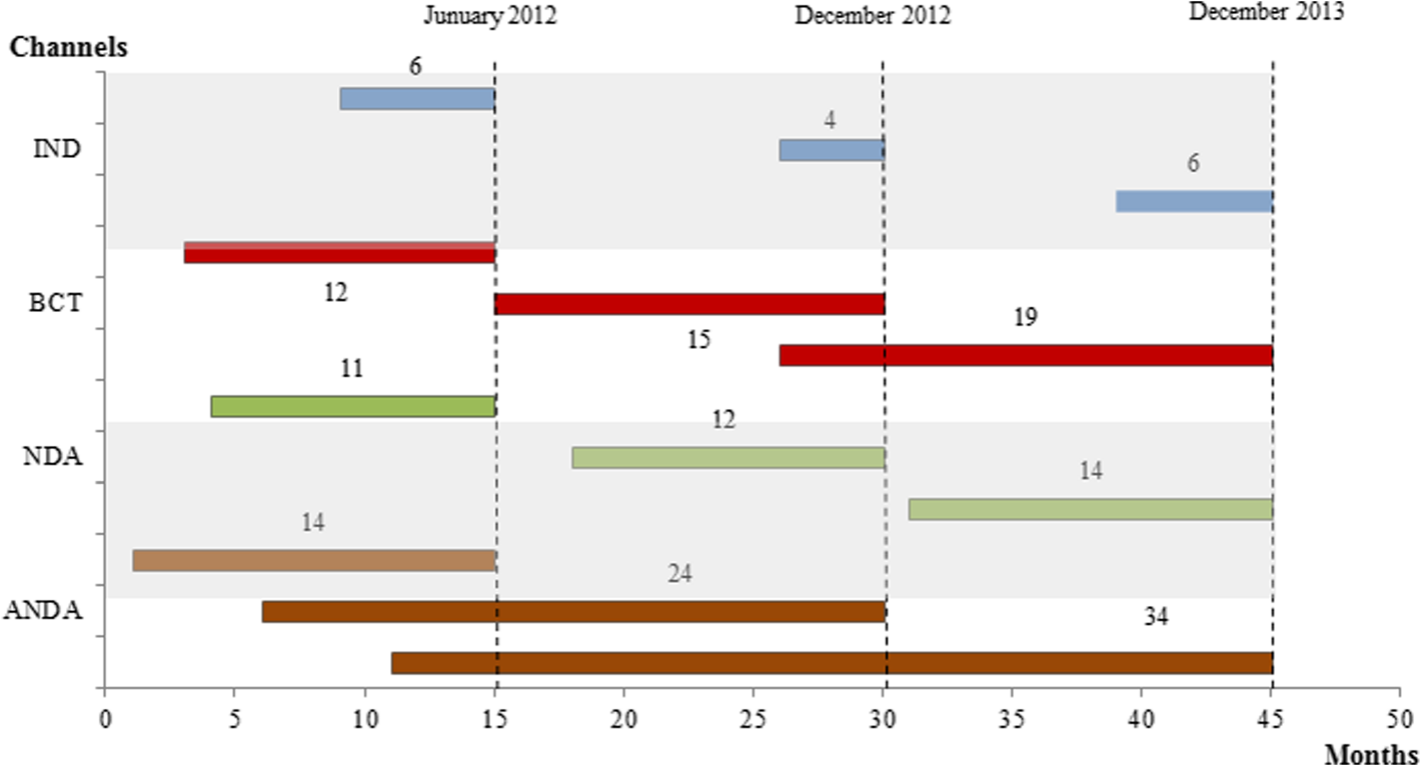
Average waiting time for technical review of chemical drugs. 1. Data source: 2013 China Drug Review Annual Report. 2. Figure 6 describes the average waiting time for technical review of chemical drugs in four channels, including Investigational New Drug (IND), New Drug Application (NDA), bridging clinical trial (abbreviated as BCT in Fig. 1) and Abbreviated New Drug Application (ANDA). Waiting time is measured in month and calculated as the difference between CDE’s reception date (the day CDE receives drug evaluation request of certain applications from CFDA) and technical review starting time. The January 2012, December 2012 and December 2013 are three time points that CDE commences technical review of certain applications

The regulatory system of pharmaceutical products in China has also contributed to the sub-development of drug innovation in China. Firstly, due to insufficient manpower of the Center for Drug Evaluation (CDE) and excessive applications of generic drug products, the drug approval time in China was often prolonged which greatly discouraged pharmaceutical R&D. The average waiting time for standard reviews was 12.3 months (see Fig. 6) which could be prolonged much further to a point of having an uncertain time for obtaining final approval [26]. In contrast, for the Food and Drug Administration (FDA) in the U.S., the New Drug Application (NDA) usually took 12.9 months after standard reviews to receive an approval [27].

On the other hand, regulatory standards in China were not consistent with international practices. As China did not join the International Conference on Harmonization of Technical Requirements for Registration of Pharmaceuticals for Human Use (ICH), innovative drugs which already marketed in other countries had to undergo the new drug registration pursuant to China’s Drug Registration Regulation. Consequently, the entry of import drugs to the local market could be delayed as many as 7 years on average compared with the date the drug first marketed in other countries [28]. For example, Gardasil (Human papilloma virus (HPV) vaccines), which is used to prevent infections by certain types of human papillomavirus, has been first marketed by MSD company in 2006. However, this widely-used vaccine that has been marketed in more than 130 countries and regions in the world, has not yet approved by CFDA. Furthermore, for registration purpose, it was necessary to repeat the clinical trials of import drugs in China as the China’s Good Clinical Practice (GCP) was different from the GCP according to ICH. In addition, pre-approval by the CFDA was needed before clinical trials could be conducted, which meant another several months or more waiting time. The international clinical trial multi-center might offer some advantages as a quick channel for import drugs but this only applied to drugs that were already marketed or at least entered phase II clinical trial in other countries [29]. As a result, simultaneous global development of drugs faces great challenges in China.

Finally, unlike the practice of marketing authorization holder (MAH) widely adopted in many developed countries, drug marketing authorization in China was only granted to pharmaceutical manufacturers with production authorization. This created significant threat to the initiative of technology transfer between R&D players and pharmaceutical firms. On one hand, R&D institutions might lack the manufacturing facilities and thus were not eligible for applying marketing approval of the drug developed in-house. On the other hand, drug manufacturers needed to shoulder the pressure of massive financial investment for every new production line when developing a new product. The potential risk caused by overcapacity would further constrain the future development of enterprises or even the entire pharmaceutical sector.

### Finance and service institutions

As a major component of innovation system, financing system firstly poses significant challenges to drug innovation in China. Improper funds arrangement was common and usually resulted in inefficiency of new drug R&D. Public investment was the key funding source for R&D institutes in the pharmaceutical sector, of which more than 81% R&D expenditure was accounted for with government funding while private investment only accounted for 5.41% in 2012 [30]. Although the central government had allocated increasing resources into R&D institutions in recent years, investment for basic research was insufficient. In China, only 4.7% of R&D investment was used to improve basic research which was little compared with the figure in some developed counties (see Table 1). This was especially problematic for pharmaceutical industry as preliminary research was the source of new ideas important for fueling subsequent innovation and had significant impact on the performance of new drug discovery [31].

**Table 1:** International comparison of R&D expenditure

By types of Research %	China	USA	Japan	France	Australia	South Korea	Russian
	(2011)	(2009)	(2009)	(2009)	(2008)	(2010)	(2010)
Basic Research	4.7	19.0	12.5	26.0	20.0	18.2	19.6
Applied Research	11.8	17.8	22.3	39.8	38.6	19.9	18.8
Experimental Development	83.5	63.2	60.5	34.2	41.4	61.8	61.6

Data source: China Statistical Yearbook on Science and Technology

For new drug developers, contributions of venture capital (VC) were limited in China. In particular, the SMEs considerably relied on government investment to support their innovation projects [18]. Since VC market only started 30 years ago, VC activity and investment level in the pharmaceutical sector was substantially lower in China than in other developed counties. According to S&P Capital IQ estimates, 711 VC and private equity (PE) funds had life sciences investments in the U.S., whereas only 89 similar funds in China. Moreover, out of the 89 funds, only 19 made more than one investment [32]. There were also other issues about financing for drug innovation. For instance, lack of an efficient investment exit channel made it difficult for investors to withdraw capital gains. As a result, a lot of VC only paid attention to short-term and less innovative projects [7]. Volatility of stock markets, highly exaggerated price to earnings ratios, and lack of sophisticated secondary markets were also detrimental to the financing for high-risk new drug R&D projects [18, 33].

At last but not least, barriers often cited in the literature were also found to be the key factors influencing drug innovation in China which included lack of practical and effective IP (intellectual property) protection and enforcement strategies [34], growing of counterfeit and substandard medicines, and undeveloped technology transaction platform and intermediary agencies.

## Opportunities for China’s pharmaceutical innovation

As two sides of the same coin, China’s pharmaceutical innovation still has various unique opportunities, despite of so many obstacles mentioned above. For instance, during the stage of the “Key Drug Innovation Project” from 2009 to 2011, 62 NDAs originated from this project were approved by the CFDA and about 400 categories entered the clinical research stage [35, 36]. Moreover, some positive efforts have been made in recent years. For example, recruitment of Chinese scientists back from abroad, China is embracing ‘Thousand Talents plan’ [37]. The latest news reported that The Chinese Academy of Sciences (CAS), the heart of China’s scientific development, is making unprecedented structural reforms to foster collaboration and to turbocharge research [38]. The CFDA issued a draft amendment to the Drug Registration Regulation, and is planning to revise Drug Administration Law of China comprehensively. The article further analyzes comparative advantages of China’s drug innovation system in the global context, elaborated one-by-one as below.

### Growing pharmaceutical market

**Fig. 7:**
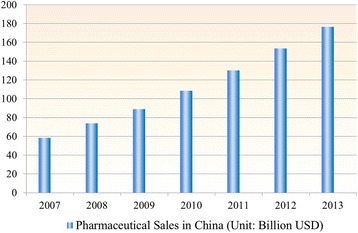
Pharmaceutical Sales in China from 2007 to 2013. 1. Data source: 2014 China Pharmaceutical Market Development Bluebook (Southern Medicine Economic Institute) 2. Exchange rate: USD/RMB = 1/6.2291

The pharmaceutical market in China will continue to grow for multiple reasons. The trend of ‘globalization’ in healthcare industry accompanied by an increased needs for better medications in developing countries are clear [39]. Also, the pharmaceutical market in China is expected to see robust growth (see Fig. 7).

Nationally, as home to nearly 20% of the world’s population, the senior population (over 65 years) in China will be expected to be 9.7% in 2016 [40]. Together with economic growth and more healthcare awareness, higher demand for health care services including pharmaceutical products can be expected. Moreover, the Chinese government is prepared to put in $136 billion USD to develop the national healthcare system and to enhance the Basic Medical Insurance (BMI) coverage from approximately 65% of the population to 90%. China’s healthcare expenditure will have been rising more rapidly [40].

The dramatic growth of healthcare demand and expenditure in China implies tremendous market opportunities in near future. For example, the prevalence of diabetes in China escalated from 0.9% in 1980 to 11.6% in 2010 [41], and China has the largest number of diabetes sufferers in the world at more than 96 million [42]. Currently, treatments for diabetes patients in China cost around $ 2.7 billion each year, and the cost will continue to increase. Consequently, all these trends are favorable to significantly drive the development of innovation.

### Increasing R&D funding

**Fig. 8:**
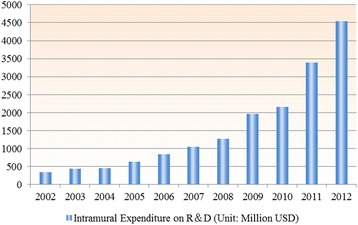
Intramural Expenditure on R&D in Chinese pharmaceutical industry. 1. Data Source: China Statistical Yearbook on High Technology Industry. 2. Exchange rate: USD/RMB = 1/6.2291

The R&D investment is considered as crucial fuels to catalyze innovation. Consequently, the dramatic growth of R&D investment in China generates enormous momentum to pharmaceutical R&D activity. On the economic recession background, many developed countries have reduced the budget on drug R&D. The U.S. cut down R&D expenditures from 38% of the global total in 1999 to 31% in 2009 [6]. In contrast, China showed the largest percentage increase of R&D investment in the world (see Fig. 8).

In pharmaceutical sector, in order to create an innovation-oriented environment, the China government will increase the drug innovation funding by launching appropriate projects. For instance, the ‘Key Drug Innovation project’ launched in 2007 was a notable example. During the entire 12th Five- Year Plan, the project ‘Key Drug Innovation’ was supported with about $16 billion USD from the central government and more than $49 million USD from local governments [7]. As the second largest R&D performer, comparison of the global compound annual growth rate of biomedical R&D expenditures by country, China showed the most rapid rise, from approximately $2.0 billion in 2007 to over $8.4 billion in 2012 with a compound annual growth rate of 32.8% [43].

### Distinctive R&D source

China’s major advantage in life science is the distinctive R&D source in terms of large patient samples, wide disease spectrum, great biodiversity, and strong basis of traditional Chinese medicine (TCM). In 2012, there were 1431 hospitals in China, of which 420 had GCP certifications and a rich source of patient enough for multiple clinical R&D studies [44]. More importantly, distinct multiple patient populations and wide disease spectrum in China are beneficial to broaden the scope of new research activities in the healthcare system. For example, some specific diseases such as diabetes, liver cancer, stomach cancer, and neck cancer have a relatively high prevalence in Asian countries compared to the U.S. and European countries. The patient pool in China allows the development of specific knowledge such as biomarkers, genetics and therapies [45].

Meanwhile, China is one of the countries with the richest biological resources and diversities, which has approximately 10% of the world’s biological resources [46]. Additionally, with further research of active components and pharmacological mechanisms, TCM will serve the global health demands and broaden the pipeline of natural medicine discovery and development, increasing the importance of Chinese herbal medicines in therapeutic systems especially for cancer, HIV, diabetes and cardiovascular disease therapies. The most famous example is artemisinin, which is isolated from the plant Artemisia annua, sweet wormwood, an herb employed in Chinese traditional medicine. Artemisinin has been recognized by international group as a standard treatment worldwide for malaria [5].

### Increasing international involvements

The favorable conditions mentioned above have attracted more and more multinational pharmaceutical companies to China. Cost advantage related to developing health product in China has been attributed to the low-costs in scientific talent, clinical trials and raw materials available in the country, with the lowest figure estimated to be 10% of similar costs in the U.S [5]. As a result, with exception of pharmaceutical R&D outsourcing moving to China, the linkage between domestic R&D organizations and multinational corporations has been increasingly prominent in R&D activity. Meanwhile, the strategies of large-cap pharmaceutical companies are steering to emphasize more on the discovery and development of medicines for China-specific and lifestyle-associated diseases. China has become one of the top markets pursued by global pharmaceutical companies to conduct R&D activities [3]. Increasing numbers of multinational pharmaceutical companies has established their R&D headquarters in China. For instance, AstraZeneca China has its headquarters in Shanghai, with 23 branch offices in major cities across China. Pfizer’s China Research and Development Centre were established in 2005 to support global R&D by partnering with clinical research organizations, biotechnology companies and academic researchers. It is beneficial for China’s pharmaceutical innovation that these high-quality multinational pharmaceutical companies moving in China will play innovation together with local institutions and further generate spillover effects on the healthcare system [3, 47].

## Conclusions

In summary, this study addressed the barriers and opportunities for pharmaceutical innovation in China. One hand, China’s pharmaceutical sector is confronted with inevitable barriers hindering the pace of drug innovation, including academic, industrial, institutional and financial constraints. To reshape China and change the reputation of made-in-China to discovered-in-China is highly challenging. On the other hand, China exhibits unique advantages in the development of healthcare industry as shown by the dramatic growth in terms of R&D investment, healthcare expenditure and international cooperation. The increasingly intertwined relationship of both competition and cooperation in the global healthcare industry is of great significance to remove obstacles and create more opportunities for China’s pharmaceutical sector. The most important thing is, to break though innovation barriers and take advantage of the opportunities that are currently available for improving drug innovation in China, and further integrate self-advantages into global value-chain of healthcare product development. All of these will greatly facilitate the development of pharmaceutical innovation in China. As a result, China will play increasingly important role in the global innovation network, and more extensively involved in global healthcare innovation in near future.

## Abbreviations

ANDA: Abbreviated New Drug Application
APIs: Active pharmaceutical ingredients
BCT: Bridging clinical trial
BMI: Basic Medical Insurance
CAS: Chinese Academy of Sciences
CDE: Center for Drug Evaluation
CFDA: China Food and Drug Administration
FDA: Food and Drug Administration in the U.S
GCP: Good Clinical Practice
ICH: Harmonization of Technical Requirements for Registration of Pharmaceuticals for Human Use
MAH: Marketing authorization holder
NDA: the New Drug Application
PE: Private equity
R&D: Research and Development
SCI: Science Citation Index
TCM: Traditional Chinese medicine
VC: Venture capital
